# The *Trypanosoma cruzi* Diamine Transporter Is Essential for Robust Infection of Mammalian Cells

**DOI:** 10.1371/journal.pone.0152715

**Published:** 2016-04-06

**Authors:** Marie-Pierre Hasne, Radika Soysa, Buddy Ullman

**Affiliations:** Department of Biochemistry and Molecular Biology, Oregon Health & Science University, Portland, Oregon, United States of America; Univ. Georgia, UNITED STATES

## Abstract

*Trypanosoma cruzi* is incapable of synthesizing putrescine or cadaverine *de novo*, and, therefore, salvage of polyamines from the host milieu is an obligatory nutritional function for the parasite. A high-affinity diamine transporter (TcPOT1) from *T*. *cruzi* has been identified previously that recognizes both putrescine and cadaverine as ligands. In order to assess the functional role of TcPOT1 in intact parasites, *a* Δ*tcpot1* null mutant was constructed by targeted gene replacement and characterized. The Δ*tcpot1* mutant lacked high-affinity putrescine-cadaverine transport capability but retained the capacity to transport diamines via a non-saturable, low-affinity mechanism. Transport of spermidine and arginine was not impacted by the Δ*tcpot1* lesion. The Δ*tcpot1* cell line exhibited a significant but not total defect in its ability to subsist in Vero cells, although initial infection rates were not affected by the lesion. These findings reveal that TcPOT1 is the sole high-affinity diamine permease in *T*. *cruzi*, that genetic obliteration of TcPOT1 impairs the ability of the parasite to maintain a robust infection in mammalian cells, and that a secondary low-affinity uptake mechanism for this key parasite nutrient is operative but insufficient for optimal infection.

## Introduction

The protozoan parasite *Trypanosoma cruzi* is the causative agent of Chagas’ disease, a devastating malady that causes insidious and often fatal destruction of the myocardium and ion conduction systems of the heart. Neurological disorders and gastrointestinal manifestations are also common in Chagas’ disease. Six to ten million people are infected with *T*. *cruzi* in Central and South America [1, 2], and ~300,000 people in the United States are seropositive for the parasite [3–5]. *T*. *cruzi*, is transmitted to vertebrate hosts by an insect vector, collectively termed reduviid bugs, in which the parasites proliferate as non-infective epimastigotes in the gut and later develop into infective but nonreplicating metacyclic trypomastigotes. The trypomastigotes are present in the feces of the insect that are deposited on the skin of the mammalian host when the insect feeds, and the trypomastigotes eventually enter the host through the bite abrasion. Once inside the host, the metacyclic trypomastigotes invade a wide range of nucleated cells and transform into immotile intracellular amastigotes. The amastigotes eventually transform back into intracellular trypomastigotes and spread to neighboring cells upon rupture of the infected cells. The infection cycle is completed when an uninfected reduviid bug obtain a blood meal from an infected human or animal. [6].

Chagas disease consists of two distinct phases, acute and chronic. The initial, acute phase occurs post-inoculation, is generally mild or even asymptomatic, and subsides in 2–3 months. The infection then proceeds to the chronic phase, which could be asymptomatic, or in approximately 25–30% of infections, is symptomatic and in which cardiomyopathy or occasionally enlargement of the organs of the digestive track are the symptomatic hallmarks. [2]. The current arsenal of drugs available for the treatment of Chagas disease is limited, and the two most commonly used agents, benznidazole and nifurtimox, are toxic and only somewhat effective against the chronic and sinister stage of the disease [7]. Consequently, there is a critical need for more efficacious drugs and for the validation of new drug targets that are either unique to *T*. *cruzi* or dissimilar to their human counterparts. The polyamine pathway is a striking example of a metabolic disparity between *T*. *cruzi* and its mammalian host.

Polyamines are aliphatic polycations that play key roles in a variety of fundamental cellular processes including growth, differentiation, and macromolecular biosynthesis, although most of the precise biological roles of polyamines remain elusive [8, 9]. Human cells accommodate three polyamines, putrescine, spermidine, and spermine, all of which are derived from the basic amino acids arginine and ornithine, while *T*. *cruzi* includes a fourth, the diamine cadaverine, a decarboxylated derivative of lysine [10, 11]. The polyamine biosynthetic pathway has been targeted in anti-trypanosomal drug-therapies, in particular for the treatment of African sleeping sickness caused by *Trypanosoma brucei*, a parasite in the same genus as *T*. *cruzi*. α-difluoromethylornithine (DFMO), a suicide inhibitor of ornithine decarboxylase (ODC), can eradicate *T*. *brucei* infections in mice [12] and has been successfully deployed in the field for the treatment of African sleeping sickness [13–15]. The validity of the polyamine pathway as an antitrypanosomal target is reinforced by the observations that 5'-{(Z)-4-amino-2-butenylmethylamino}-5'-deoxyadenosine (MDL 73811) and its 8-methyl derivative (Genz-644131), irreversible inhibitors/inactivators of S-adenosylmethionine decarboxylase (ADOMETDC) [16, 17], are effective *in vitro* against *T*. *b*. *rhodesiense* and *T*. *b*. *brucei* and can realize cures of both subspecies in mice at concentrations two orders of magnitude less that of DFMO [18–22]. Thus, the polyamine pathway has been targeted for both experimental and human *T*. *brucei* infections, and validation of its counterpart in *T*. *cruzi* is a logical upshot of these findings [18, 23].

*T*. *cruzi* expresses an unusually austere polyamine biosynthetic pathway that, unlike the human equivalent, consists of three or possibly four components: ADOMETDC, prozyme, spermidine synthase (SPDSYN), and possibly a spermine synthase (SPMSYN) [24, 25]. Conspicuously, *T*. *cruzi* lacks both arginase (ARG) and ODC activities [10, 26], the first two enzymes of the polyamine biosynthetic pathway of mammals and other polyamine prototrophs such as *T*. *brucei* [11, 27], and no *ARG* or *ODC* homolog can be found in the annotated *T*. *cruzi* genome [28]. Thus, *T*. *cruzi*, unlike mammals, *T*. *brucei*, and other polyamine synthesizing organisms, is unconditionally reliant on the acquisition of polyamines from the host milieu for survival and growth.

Salvage of host putrescine is mediated by a high affinity polytopic membrane transporter, TcPOT1 also denominated as TcPAT12, which was first functionally identified at the molecular level from the hybrid CL Brener strain of *T*. *cruzi*, the genome reference strain [29, 30]. The *TcPOT1* locus consists of two alleles, one from each *T*. *cruzi* haplotype, and both, *TcPOT1*.*1* and *TcPOT1*.*2*, encode a functional diamine transporter that recognizes both putrescine and cadaverine with high affinity [29]. A biochemically discrete spermidine transport activity has also been detected in *T*. *cruzi* epimastigotes but has yet to be identified at the molecular level [31].

The function of TcPOT1 within a cellular context, however, has not been heretofore assessed. To test the functional role of TcPOT1 in intact *T*. *cruzi*, a Δ*tcpot1* null mutant was created by double targeted gene replacement and characterized with respect to diamine transport capability and ability to cause infection in mammalian cells. The Δ*tcpot1* knockout lacked high affinity putrescine and cadaverine transport capacity but was still capable of taking up spermidine and arginine at wild type rates. Furthermore, a low affinity putrescine uptake capacity could be detected in the Δ*tcpot1* background. The Δtcpot1 mutant displayed altered polyamine pools and a markedly reduced capacity to infect Vero cells. These data reveal that TcPOT1.1 is the sole high affinity putrescine-cadaverine transporter in *T*. *cruzi* and that genetic interruption of the polyamine acquisition pathway in *T*. *cruzi* can impact parasite infectivity. The findings bolster the legitimacy of the polyamine pathway as a prospective antitrypanosomal target for Chagas disease.

## Materials and Methods

### Chemicals and reagents

[1,5-^14^C] Cadaverine dihydrochloride (15.8 mCi mmol^-1^) and [2,3-^3^H] putrescine dihydrochloride (80 Ci/mmol) were purchased from Moravek Biochemicals (Brea, CA), [^3^H(N)] spermidine trihydrochloride (32.35 Ci/mmol) was bought from PerkinElmer (Boston, MA), and [α-^32^P]dCTP was acquired from MP Biomedicals (Irvine, CA). The plasmids pDONR^™^221, pDONR-P4P1R, pDONR P2R-P3R, and pDEST^™^ R4-R3 were obtained from Invitrogen Corp. (Carlsbad, CA). Oligonucleotides were synthesized by Invitrogen Corp., and *Pfu ultra* high fidelity polymerase DNA polymerase AD was from Agilent Technologies (La Jolla, CA). Vero cells CCL-81^™^ were purchased from ATCC (Manassas, VA). T. cruzi CL brener epimastigotes were obtained from Dr. Landfear. Fetal calf, horse, and chicken sera were procured from HyClone (ThermoFisher Scientific, Logan UT). All other materials and reagents were of the highest quality commercially accessible.

### Cell culture

*T*. *cruzi* epimastigotes from the hybrid CL Brener strain were grown at 28°C in liver infusion tryptose (LIT) broth [32] that was supplemented with 10% heat-inactivated FBS or in SDM-79 medium supplemented with 7.5 mg/ml hemin and 10% heat-inactivated, dialyzed chicken serum. Metacyclic trypomastigotes were harvested from stationary phase cultures and selected by complement lysis [33] in Roswell Park Memorial Institute medium (RPMI) medium supplemented with 30% horse serum. Vero cells, a kidney epithelial cell line derived from African green monkey, were propagated at 37°C in 5% CO_2_ in RPMI medium supplemented with 10% FBS. Tissue culture-derived trypomastigotes were obtained after cell lysis of Vero cell monolayers that had been infected with metacyclic trypomastigotes [34].

### Generation of gene targeting constructs

Two drug resistance cassettes for targeting the two *TcPOT1* alleles were constructed using the MultiSite Gateway^®^ (MultiSite Gateway^®^ Three-Fragment Vector Construction Kit, Invitrogen) following the manufacturer’s instructions. The open reading frames of the neomycin and hygromycin phosphotransferase genes flanked at their 3’-termini by the 3’-untranslated region (UTR) of the glyceraldehyde-3-phosphate dehydrogenase (*GAPDH*) gene were amplified by PCR from the pTEX-Neo and pTEX-Hyg vectors [35, 36], respectively, using the *att*B-attached primers listed in S1 Table. The two PCR products were gel-purified and cloned separately into the pDONR^™^221 plasmid. These chimeric plasmids were designated pENTR-*Neo-*3’-UTR*GAPDH* and pENTR-*Hyg*-3’-UTR-*GAPDH*, respectively (S1 Fig). The 5’ and 3’ UTRs of *TcPOT1*.*1* and *TcPOT1*.*2* were PCR-amplified from *T*. *cruzi* CL Brener genomic DNA using primers introducing *att*B sites into the amplified DNAs (S1 Table). The PCR products were gel-purified, and the 5’ UTRs of *TcPOT1*.*1* and *TcPOT1*.*2* were cloned into the pDONR-P4P1R vector, while the 3’ UTRs were cloned into the pDONR P2R-P3R plasmid (S1 Fig). These constructs were specified as pENTR-5’UTR*TcPOT1*.*1*, pENTR-3’UTR*TcPOT1*.*1*, pENTR-5’UTR*TcPOT1*.*2 and* pENTR-3’UTR*TcPOT1*.*2*, according to the *TcPOT1* allele and corresponding flanking region. The assembly of the two final gene targeting plasmids involved ligation of pENTR-5’UTR*TcPOT1*.*1*, pENTR-*Neo*-3’-UTR-*GAPDH*, and the pENTR-3’UTR*TcPOT1*.*1* in a lambda recombination reaction into the destination plasmid pDEST^™^ R4-R3 to generate the final construct designated pDEST-5’UTR*TcPOT1*.*1*-*Neo*-3’UTR*GAPDH*-3’UTR*TcPOT1*.*1* and ligation of pENTR-5’UTR*TcPOT1*.*2*, pENTR-*Hyg*-3’-UTR-*GAPDH*, and pENTR-3’UTRTcPOT1.2 into a second lambda recombination reaction to create pDEST-5’UTR*TcPOT1*.*2*-*Hyg*-3’UTR*GAPDH*-3’UTR*TcPOT1*.*2* (S1 Table). The fidelity of the two constructs was confirmed by automated DNA sequencing.

### Parasite transfections

Early log phase *T*. *cruzi* epimastigotes grown in LIT medium were collected by centrifugation, washed once in 21 mM HEPES, 137 mM NaCl, 5 mM KCl, 0.7 mM Na_2_HPO_4_, and 6 mM glucose, pH 7.5 (HBS buffer) and resuspended at 1 X 10^8^ cells/ml in HBS buffer. For targeting the *TcPOT1*.*1* and *TcPOT1*.*2* alleles, respectively, ~10 μg of the pDEST-5’UTR*TcPOT1*.*1*-*Neo*-3’UTR*GAPDH*-3’UTR*TcPOT1*.1 and pDEST-5’UTR*TcPOT1*.*2*-*Hyg*-3’UTR*GAPDH*-3’UTR*TcPOT1*.*2* targeting plasmids were linearized with PvuI and AlwN1, added to 4 X 10^7^ parasites, mixed gently, and transferred to 4 mm Electroporation cuvettes plus^™^ (BIO-RAD, Hercules, USA). The cells were electroporated twice at 1500 V and 25 μF using a Gene Pulser XCell^™^ electroporator (BIO-RAD, Hercules, USA). After electroporation, cells were transferred to a tissue culture flask containing 5 ml LIT medium and 10% FBS and incubated for 24 hours prior to the addition of either G418 or hygromycin as appropriate. G418 and hygromycin concentrations employed were 200 μg/ml and 600 μg/ml respectively. Transfectants growing in bulk cultures were cloned by limiting dilution in 96 well plates using 100 μl of culture medium per well.

### Southern blot analysis

Genomic DNA was extracted from mid-exponential phase wild type, *tcpot1*.*1*::*neo*/*TcPOT1*.*2* (*tcpot1*.*1/TcPOT1*.*2*) *and tcpot1*.*1*::*neo* /*tcpot1*.*2*::*hyg* (Δ*tcpot1*) epimastigotes using a DNeasy kit (Qiagen, Hilden, Germany). The genomic DNA was digested with AflII and ApaI, and 6 μg of the digested DNA was electrophoretically separated on a 0.8% agarose gel and transferred to a nylon membrane (ManaGraph 0.22 micron, Osmonics, Minnetonka, MN) using standard protocols. The gels were probed with either the *TcPOT1*.*1* ORF or the 5’-untranslated region of *TcPOT1*.*1* that were radioactively labeled with [α-^32^P]dCTP using the RAdPrime DNA labeling system (Invitrogen, Carlsbad, CA).

### Genetic complementation of the *Δtcpot1* cell line

The Δ*tcpot1*[p*TcPOT1*.*1*::*GFP*] “add-back” line was created from the *Δtcpot1* null mutant by transfecting the knockout with a modified pTEX-*TcPOT1*.*1*::*GFP* vector [29] in which the neomycin phosphotransferase marker had been replaced with the phleomycin resistance gene from *Streptoalloteichus hindustanus* [37]. The electroporation conditions for generating the “add-back” in the null chromosomal background were identical to those described above for the generation of the knockout. The “add-back” was then selected for growth in 800 ug/ml phleomycin.

### Transport assays

*T*. *cruzi* epimastigotes in mid-log cultures were harvested and washed three times in phosphate buffered saline (PBS) supplemented with 10 mM glucose. Transport assays were carried out according to a previously published oil stop technique (Hasne and Barrett, 2000), and the incorporated radioactivity was measured by liquid scintillation spectrometry using a Beckman LS6500 scintillation counter. The data were analyzed using the Grafit software package (Erithacus Software Limited, Horley, U.K.).

### Growth Phenotype Determinations

The abilities of wild type and Δ*tcpot1* parasites to utilize different polyamines as nutrients for growth were compared. Parasites were washed three times in PBS and resuspended in SDM-79 medium supplemented with 10% dialyzed chicken serum. The parasites were seeded at 3 X 10^6^ cells/ml in the absence or presence of either 200 μM putrescine, cadaverine, spermidine, or spermine. Parasite cell densities were enumerated visually every 2 days by hemacytometer. At days 6 and 12, parasites were diluted back to 3 X 10^6^ cells/ml in order to maintain exponential growth.

### High Performance Liquid Chromatography

Putrescine, cadaverine, spermidine, and spermine pools were measured by high performance liquid chromatography (HPLC) in wild type and Δ*tcpot1* mid-exponential phase epimastigotes grown in SDM-79 medium supplemented with 10% dialyzed chicken serum using a standardized protocol [11]. 1 X 10^8^ cells were collected by centrifugation and cell extracts acid-hydrolyzed prior to derivatization with dansyl chloride. The dansylated-polyamines were separated on a Bio-Sil^®^ C18 reverse phase chromatographic column (Biorad, CA) fitted to an Agilent 1100 HPLC system and coupled to a fluorescence detector as reported [38]. The dansylated polyamines eluted from the column were quantified by fluorimetry using an excitation wavelength of 340 nm and an emission wavelength of 515 nm. 1,7-diaminoheptane was added to each of the cell pellets as an internal standard. Protein concentrations were quantified colorimetrically using the Bradford protein assay [39].

### Parasite infectivity assays

Vero cells were plated in 6-well plates fitted with square coverslips at a cell density of 1 X 10^4^ cells per well and irradiated for 20 minutes at 145.7 rad/minute (JL Shepard and Associates Mark I model 68 irradiator containing a ^137^Cs source) in order to prevent replication and overgrowth during the course of the three day infectivity experiments. Irradiated Vero cells were infected with 5 X 10^4^ trypomastigotes overnight after which the Vero cells were washed 3 times in RPMI-10% FBS and the infection was allowed to proceed for 72 hours. Cells were stained with 5 μg/ml CellMask^™^ stain (Molecular Probes, Eugene, OR) for 5 minutes at 37°C to delineate Vero cell boundaries and highlight the few remaining extracellular parasites. After removal of the staining solution, the Vero cells were fixed with 3.75% formaldehyde in PBS for 10 minutes at 37°C. Formaldehyde-fixed cells were rinsed twice in PBS, stained with 5 μg/ml 4′,6-diamidino-2-phenylindole (DAPI) to visualize Vero cell and parasite DNA, rinsed once in PBS, and mounted in SlowFade^®^ Gold antifade reagent (Molecular Probes, Eugene, OR). The percentage of infected Vero cells in a sample of 200 cells was determined on a Zeiss Axiovert 200 M microscope by visual inspection and the quantity of intracellular parasites in each infected Vero cell enumerated. Images of infected Vero cells were captured on an AxioCam MRm camera and processed using Axiovision Release 4.6. software.

## Results and Discussion

### Generation of *Δtcpot1* null mutant

**Fig 1:**
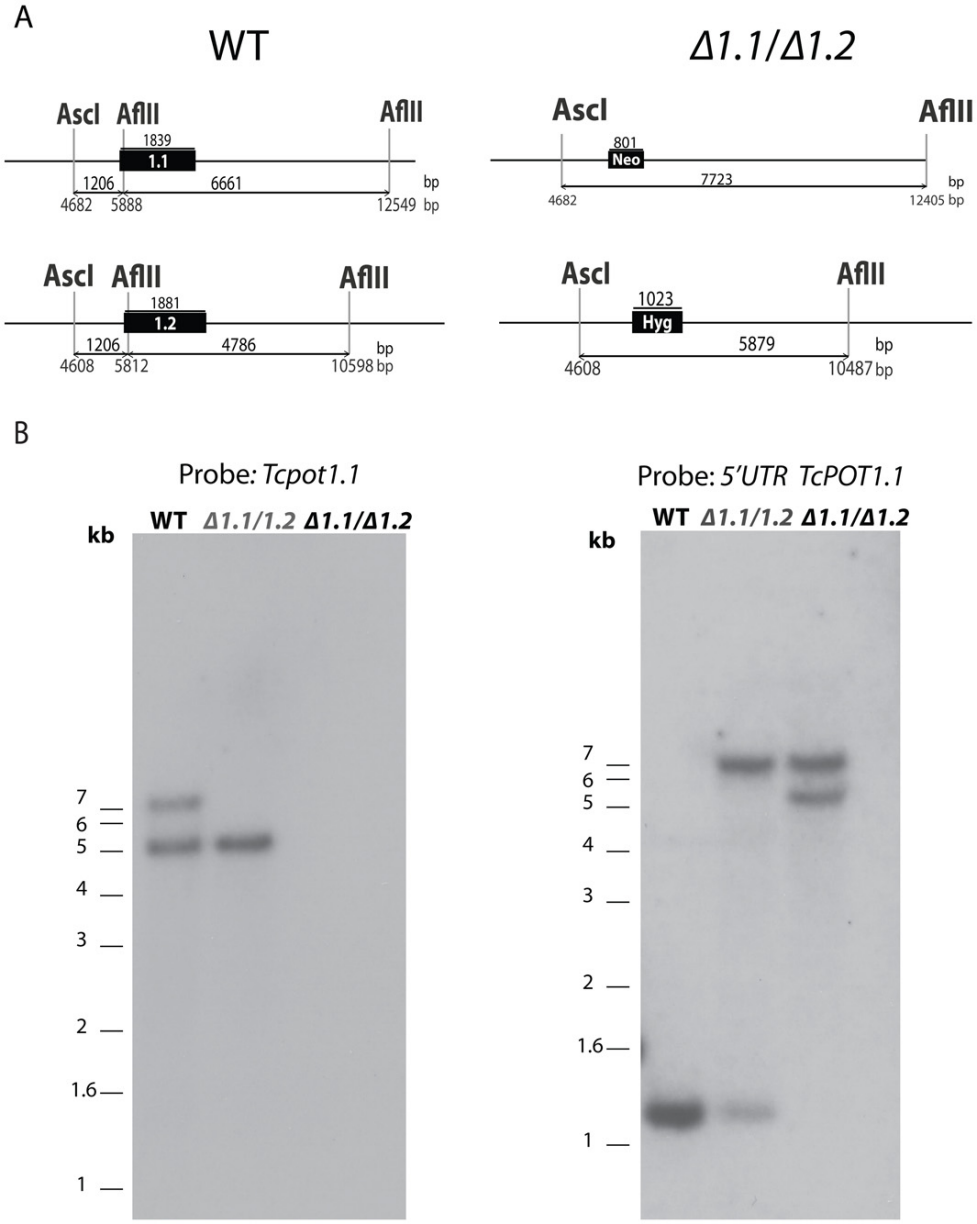
Gene targeting constructs and Southern blot analysis of *T*. *cruzi* CL Brener genomic DNA from wild type and transgenic *T*. *cruzi*. (A) Diagram of the *TcPOT1*.*1* (1.1) and *TcPOT1*.*2* (1.2) loci in wild type (WT) *T*. *cruzi* (left panel) and in the Δ*tcpot1* (*Δ1*.*1/Δ1*.*2*) null mutant after the recombination events and integration of the G418 and hygromycin drug resistance cassettes, respectively (right panel). The positions of the AflII and AscI restriction sites that are germane to the Southern blot analysis are depicted with the relevant base pair (bp) distances between them shown below the locus depiction. (B) Southern blot analysis of wild type (WT), *tcpot1*.*1*/*TcPOT1*.*2* (*Δ1*.*1/1*.*2*) and Δ*tcpot1* (*Δ1*.*1/Δ1*.*2*) cell lines digested with AflII and AscI. The blot was probed with the *TcPOT1*.*1* open reading frame (left panel) or the *TcPOT1*.*1* 5’-untranslated region (right panel).

To address the functional role of the high-affinity diamine transporter TcPOT1 in polyamine homeostasis and parasite infectivity, a Δ*tcpot1* cell line lacking TcPOT1 was generated in *T*. *cruzi* epimastigotes by double targeted gene replacement. To generate the *T*. *cruzi* Δ*tcpot1* null mutant, the *TcPOT1*.*1* and *TcPOT1*.*2* alleles were sequentially replaced with drug resistance cassettes. A *tcpot1*.*1*/*TcPOT1*.*2* heterozygote was created from wild type *T*. *cruzi* after transfection with linearized pDEST-5’UTR*TcPOT1*.*1*-*Neo*-3’UTR*GAPDH-3’UTRTcPOT1*.*1*, selected and cloned by limiting dilution in culture medium supplemented with 200 μg/ml G418. After appropriate preliminary genotyping by PCR, the *tcpot1*.*1*/*TcPOT1*.*2* heterozygote was subjected to a second round of transfection using the targeting construct derived from pDEST-5’UTR*TcPOT1*.*2*-*Hyg*-3’UTR*GAPDH*-3’UTR*TcPOT1*.*2* (Fig 1A). Southern blotting was then used to confirm the genotypes of the wild type, *tcpot1*.*1*/*TcPOT1*.*2* heterozygote, and *tcpot1*.*1*/*tcpot1*.*2* (Δ*tcpot1*) homozygous knockout. Genomic DNA digested with AflII and AscI and probed with the *TcPOT1*.*1* ORF or *TcPOT1*.*1* 5’-untranslated region revealed the expected hybridization signals for the three cell lines and confirmed the proper integration of the neomycin and hygromycin drug resistance cassettes into the parasite genome (Fig 1B). The Δ*tcpot1* mutation was not lethal to the epimastigote, despite the intrinsic diamine auxotrophy of *T*. *cruzi* [10, 26] since the null mutant could be propagated continuously.

### Polyamine transport

**Fig 2:**
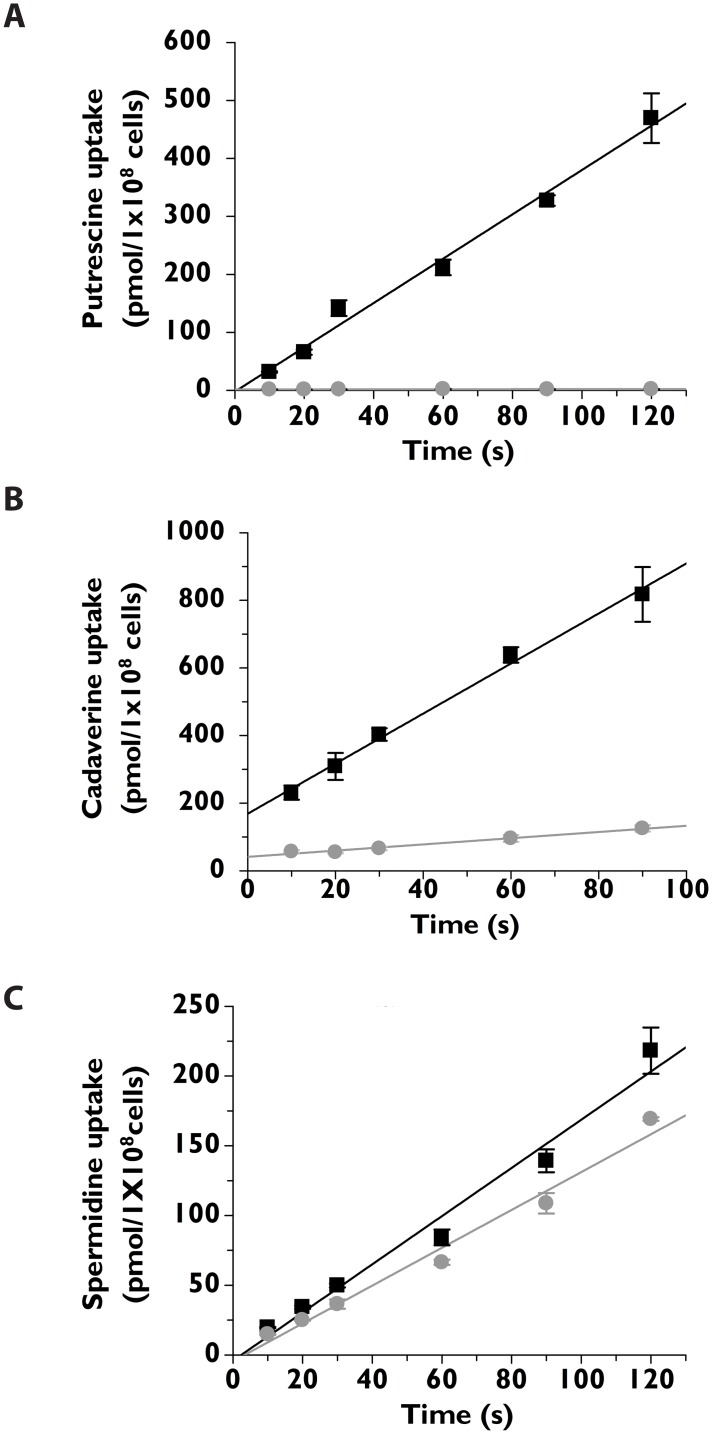
Diamine and polyamine transport by wild type and Δ*tcpot1* epimastigotes. The abilities of wild type (■) and Δ*tcpot1* (grey dot) epimastigotes to transport 1 μM (A) [^3^H]putrescine, (B) 9 μM [^14^C]cadaverine, and (C)1 μM [^3^H]spermidine over a 2 minute time course is depicted. Each graph is that of a single experiment that was performed in triplicate.

The capacity of the *T*. *cruzi* Δ*tcpot1* cell line to transport 1 μM putrescine, cadaverine, or spermidine under assay conditions that were effectively linear with time was quantified and compared to that of wild type *T*. *cruzi* epimastigotes (Fig 2). Putrescine and cadaverine transport capacity in the Δ*tcpot1* null mutant was markedly compromised (Fig 2A and 2B), while spermidine (Fig 2C) and arginine (data not shown) transport was similar to that of wild type *T*. *cruzi*.

**Fig 3:**
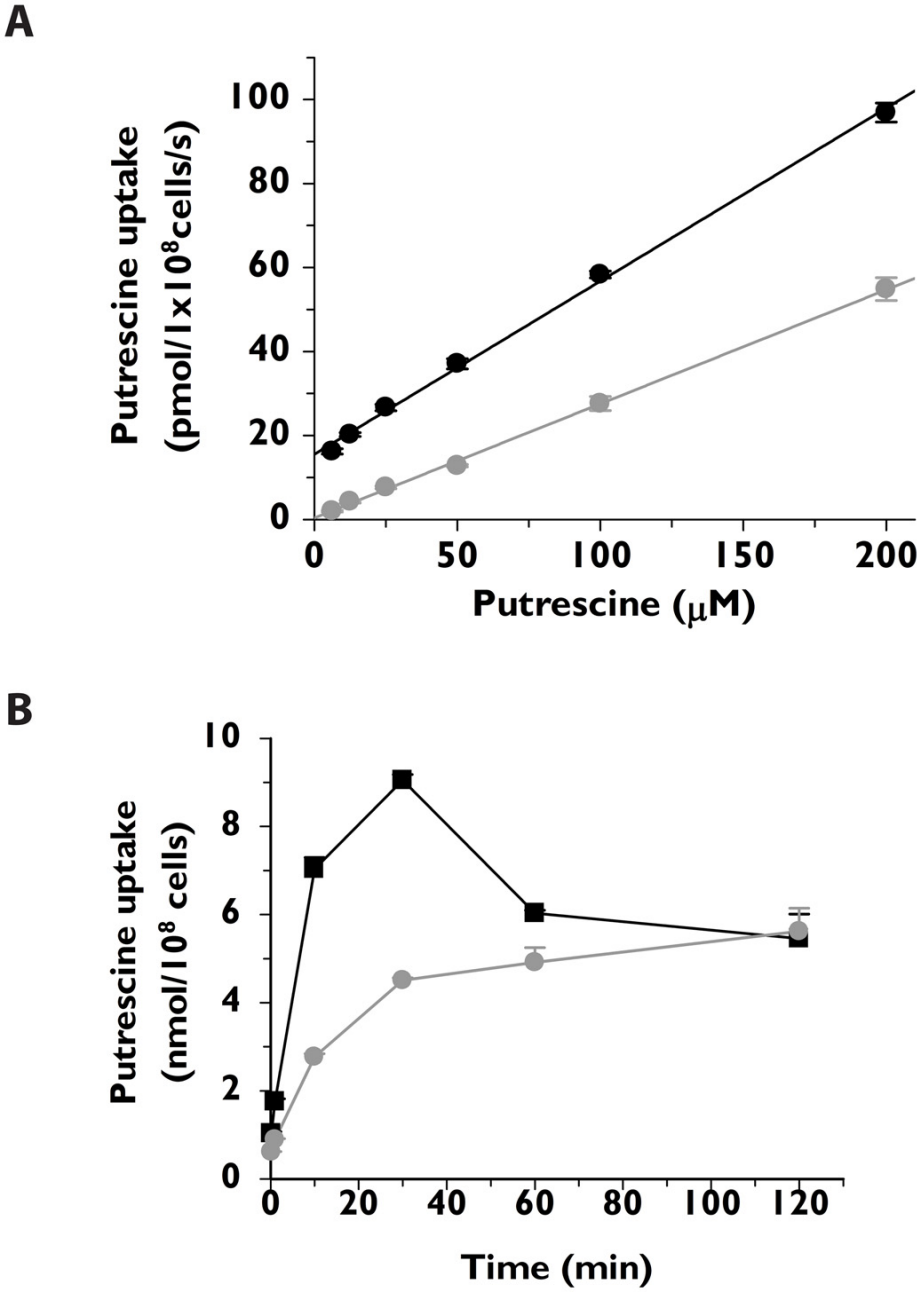
Comparison of the abilities of wild type and Δ*tcpot1 T*. *cruzi* to take up putrescine as a function of concentration and time. (A) The rates by which wild type (●) and Δ*tcpot1* (grey dot) epimastigotes incorporate extracellular [^3^H]putrescine over a range of putrescine concentrations from 5–200 μM were determined as described in Experimental procedures. Uptake measurements were determined after a 10 s exposure to the radioactive ligand. The graph is of a typical experiment that was performed in triplicate. (B) The rates of [^3^H]putrescine uptake into wild type (■) and Δ*tcpot1* (grey dot) epimastigotes were measured at 200 μM putrescine over a 120 minute time course. The data depicted are from one of three independent experiments, all with comparable results.

Measurements of putrescine uptake as a function of putrescine concentrations up to 200 μM revealed that the Δ*tcpot1* cell line expressed a non-saturable putrescine transport capability that was also observed in wild type *T*. *cruzi* (Fig 3A). The identity of this secondary putrescine uptake component is unknown, but its non-saturable profile (up to 2 mM putrescine) implies a non-carrier-mediated uptake mechanism reminiscent of fluid phase or receptor-mediated endocytosis; a process that is known to be highly active in *T*. *cruzi* [33, 40, 41]. Comparisons of putrescine accumulation as a function of time at an extracellular concentration of 200 μM revealed that Δ*tcpot1* parasites eventually accumulated putrescine to the same extent as wild type cells. At 2 hours, the total amount of putrescine taken up by wild type and Δ*tcpot1* cells was roughly equivalent (Fig 3B).

### Polyamine requirements in wild type and *Δtcpot1* epimastigotes

**Fig 4:**
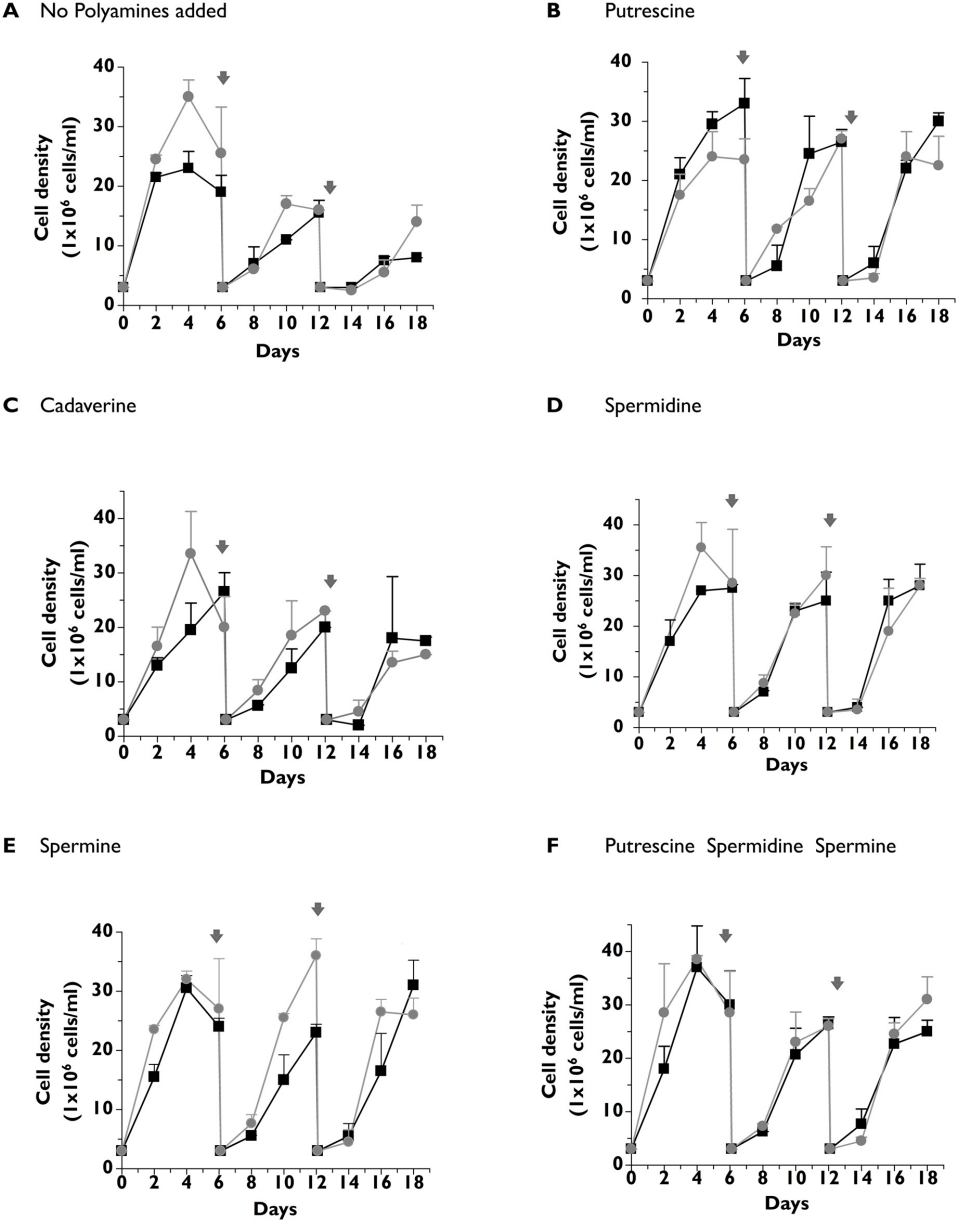
*T*. *cruzi* growth in the absence or presence of polyamines in the culture medium. The growth rates of wild type (●) and Δ*tcpot1* (grey dot) epimastigotes were determined over an 18 day time course in(A) the absence, or the presence of 200 μM concentrations of either (B) putrescine, (C) cadaverine, (D) spermidine, (E) spermine, or (F) putrescine, spermidine and spermine in combination. Parasites were seeded at 3 X 10^6^ cells ml^-1^ and cell densities assessed every 2 days. On days 6 and 12, cell cultures were diluted back to a density of 3 X 10^6^ cells ml^-1^. The dilutions are indicated in the panels by a gray arrow.

The nutritional requirements of wild type and Δ*tcpot1* epimastigotes were evaluated in medium in which the polyamine in the extracellular medium was varied. These growth tests were conducted in SDM-79 medium supplemented with dialyzed chicken serum to avoid the polyamine oxidase-catalyzed toxicity that is observed in medium containing fetal bovine serum (FBS) to which spermidine or spermine is added [42]. Wild type and Δ*tcpot1* epimastigotes grew to similar cell densities and at similar rates for the duration of the 18 day experiment when the medium was supplemented with 200 μM putrescine, spermidine or spermine (Fig 4). For both cell lines the negative impact of the absence of polyamine addition in the growing media on cell growth was observed only after several cell divisions (Fig 4A), and neither cell line grew as robustly with cadaverine as the extracellular polyamine (Fig 4C). Thus, comparisons of the abilities of wild type and Δ*tcpot1* epimastigotes to utilize various diamines and polyamines as a polyamine source revealed insignificant differences between the two lines in terms of growth rate (Fig 4), presumably because the nutritional needs of the parasite for polyamines can be satisfied by the low-affinity, high capacity diamine uptake mechanism that was uncovered in this study (Fig 3) and by biochemically distinct uptake mechanisms for spermidine [31] and, presumably, spermine.

**Fig 5:**
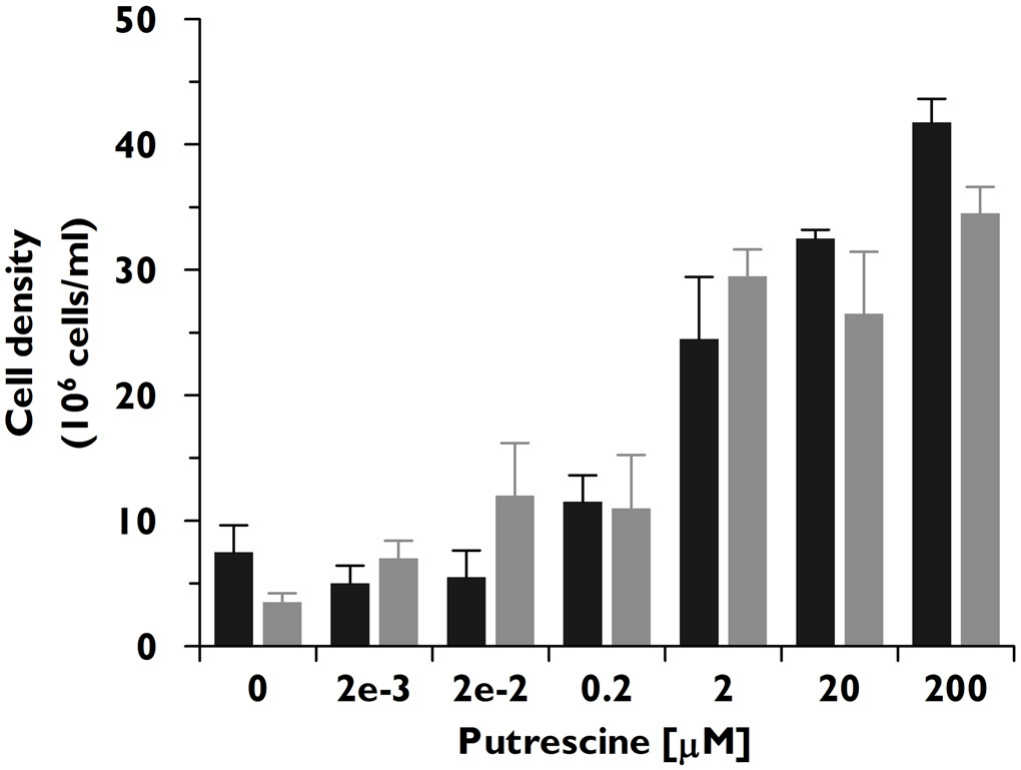
Growth of wild type and knockout parasites as a function of putrescine concentration. The growth of wild type (black bars) and Δ*tcpot1* (gray bars) epimastigotes in the absence and presence of various putrescine concentrations was assessed as described in Experimental procedures. The results presented are the averages and standard deviations of three experiments.

To assess whether the transport defect in Δ*tcpot1* could impact growth of the epimastigote in putrescine at any concentration, a dose-response curve measuring parasite cell density over a gradient of putrescine concentrations was carried out. Interestingly, similar growth profiles for wild type and Δ*tcpot1* epimastigotes as a function of putrescine levels in the extracellular milieu were demonstrated, with an optimal cell density obtained for both cell lines at a putrescine concentration in the μM range (Fig 5).

### Polyamine content in wild type and *Δtcpot1* epimastigotes

The capacity of wild type and Δ*tcpot1* epimastigotes to accumulate and interconvert polyamines was assessed by growing the two cell lines in SDM-79 culture medium lacking polyamine or supplemented with either 200 μM putrescine, cadaverine, spermidine or spermine. These experiments were also conducted in medium in which the FBS component was replaced with 10% dialyzed chicken serum in order to avoid parasite toxicity induced by polyamine oxidase that is present in FBS. Polyamine levels in wild type or Δ*tcpot1* epimastigotes grown in the absence of polyamine were below detection limits (Table 1). Wild type epimastigotes incubated with putrescine harbored comparable levels of putrescine and spermidine but contained no detectable spermine. Significantly, the Δ*tcpot1* null mutant, grown in the presence of putrescine, possessed equivalent levels of spermidine as wild type parasites, but a significantly lower (~6%) putrescine content of that of the wild type parasite. Thus, putrescine uptake is clearly rate-limiting for Δ*tcpot1* epimastigotes, but the diamine is efficiently converted to spermidine by the parasite SPDSYN. In addition, wild type *T*. *cruzi* epimastigotes accumulated significant amounts of cadaverine, while the Δ*tcpot1* parasites showed dramatically reduced cadaverine levels, ~2% of that observed in wild type parasites (Table 1). Wild type and Δ*tcpot1* parasites incubated with either spermidine or spermine contained roughly equivalent amounts of intracellular polyamine in which they were grown. Spermidine was the only polyamine detected in both lines incubated with 200 μM spermidine, whereas a small amount of spermidine was detected in cells incubated with 200 μM spermine (Table 1). Polyamine pool measurements in epimastigotes incubated with cadaverine, spermidine, and spermine showed that each accumulated within the parasite and was not extensively, if at all, metabolized to other polyamines, although a small amount of spermidine was detected in both wild type and mutant organisms after a 72 h incubation with 200 μM spermine (Table 1). This observation convincingly demonstrates that spermidine singlehandedly can satisfy the polyamine requirements of the parasite and strongly implies that spermine alone is sufficient to support growth, although a requirement for a small amount of spermidine in the spermine-grown parasites cannot be ruled out. The fact that epimastigotes incubated for three days with spermidine do not accumulate putrescine indicates that the parasite does not express the spermine/spermidine degradation pathway, i.e., spermidine/spermine *N*^*1*^-acetyltransferase and polyamine oxidase, that is operative in mammalian cells [43]. The lack of polyamine catabolic activity in *T*. *cruzi* epimastigotes is consistent with the absence of the corresponding gene homologs in the *T*. *cruzi* genome [28]. Furthermore, the lack of spermine accumulation in *T*. *cruzi* epimastigotes incubated with spermidine suggested a lack of a SPMSYN activity in the parasite, although these data contrast with those obtained previously by Hunter *et al*. [11] who demonstrated that a 6 day incubation with putrescine resulted in substantial accumulation of spermine. Whether the discrepancy in apparent SPMSYN activities in the two studies can be ascribed to strain specificity or other factors is not known, but the validation of a functional SPMSYN remains to be determined. The *T*. *cruzi* genome has no obvious *SPMSYN* homolog but appears to accommodate two *SPDSYN* paralogs (Non esmeraldo: TcCLB.510339.50 and TcCLB.510337.40 and Esmeraldo: TcCLB.504033.130 and:TcCLB.503855.20), each of which is 297 amino acids in length, 99% identical with each other, 41.7% identical with human SPDSYN, but only 12.9% identical with human SPMSYN. Thus, it is reasonable to speculate that *T*. *cruzi* possesses two *SPDSYN* paralogs. Interestingly, although the two *SPDSYN* paralogs are both located on chromosome 8, they do not appear to be tandemly repeated as are many *T*. *cruzi* genes [28], since they are separated by 36–39 kb of intergenic DNA which contains at least 8–10 additional open reading frames.

**Table 1:** Polyamine content of wild type and Δ*tcpot1 T*. *cruzi* grown in various polyamine sources.

Polyamine intracellular content (nmol/mg protein) for Wild type / Δtcpot1				
	Putrescine	Cadaverine	Spermidine	Spermine
**Additions**				
**None**	ND **/ *ND***	ND **/ *ND***	ND **/ *ND***	ND **/ *ND***
**Putrescine**	50.6 ± 14 **/ *3*.*1 ±2*.*1***	ND **/ *ND***	53.6 ± 10.5 **/ *56 ± 10***	ND **/ *ND***
**Cadaverine**	ND **/ *ND***	72.9 ± 33.0 / ***1*.*3***	ND **/ *2*.*5 ± 1*.*0***	ND **/ *ND; 1*.*6***
**Spermidine**	ND **/ *ND***	ND **/ *ND***	109.8 ± 61.3 **/ *74*.*6 ± 21*.*0***	ND **/ *ND***
**Spermine**	ND **/ *ND***	ND **/ *ND***	8.8 ± 0.3 **/ *10*.*4 ± 2*. *8***	208.7 ± 51.8 **/ *206*.*3 ± 101***

Epimastigotes were grown for 3 days in SDM-79 media and 10% dialyzed chicken serum supplemented or not by 200 μM putrescine, cadaverine, spermidine, or spermine. The diamine/polyamine content of wild type and Δ*tcpot1* (bold and italicized) cells was determined by HPLC, and the data shown are the averages and standard deviations of three different biological replicates. ND means not detected. ND; 1.6 are the results of two determinations where spermine was not detected and one when it was found.

Both the wild type and the Δ*tcpot1* null mutant grown in the absence of diamine or polyamine supplement were able to replicate through several cell divisions (Fig 4) despite the fact that polyamine levels were undetectable in polyamine-starved cells after three days (Table 1) but the method employed to measure parasites polyamine content only detects free polyamines. Stores of polyamines, either bound to macromolecules such as polyphosphates contained in the acidocalcisomes, which would not have been detected in the polyamine pool analyses, or generated from the breakdown of trypanothione, a spermidine-containing dithiol that plays a vital role in redox balance in *T*. *cruzi* [44, 45], could play a role in sustaining growth in polyamine-free medium. This observation also implies that whatever specific roles polyamine play in *T*. *cruzi*, depletion of polyamine pools does not immediately impact the ability of the parasite—at least the epimastigote form—to divide, although longer term deprivation triggers an adverse impact on growth (Fig 4).

### Infectivity of *Δtcpot1* in mammalian cells

**Fig 6:**
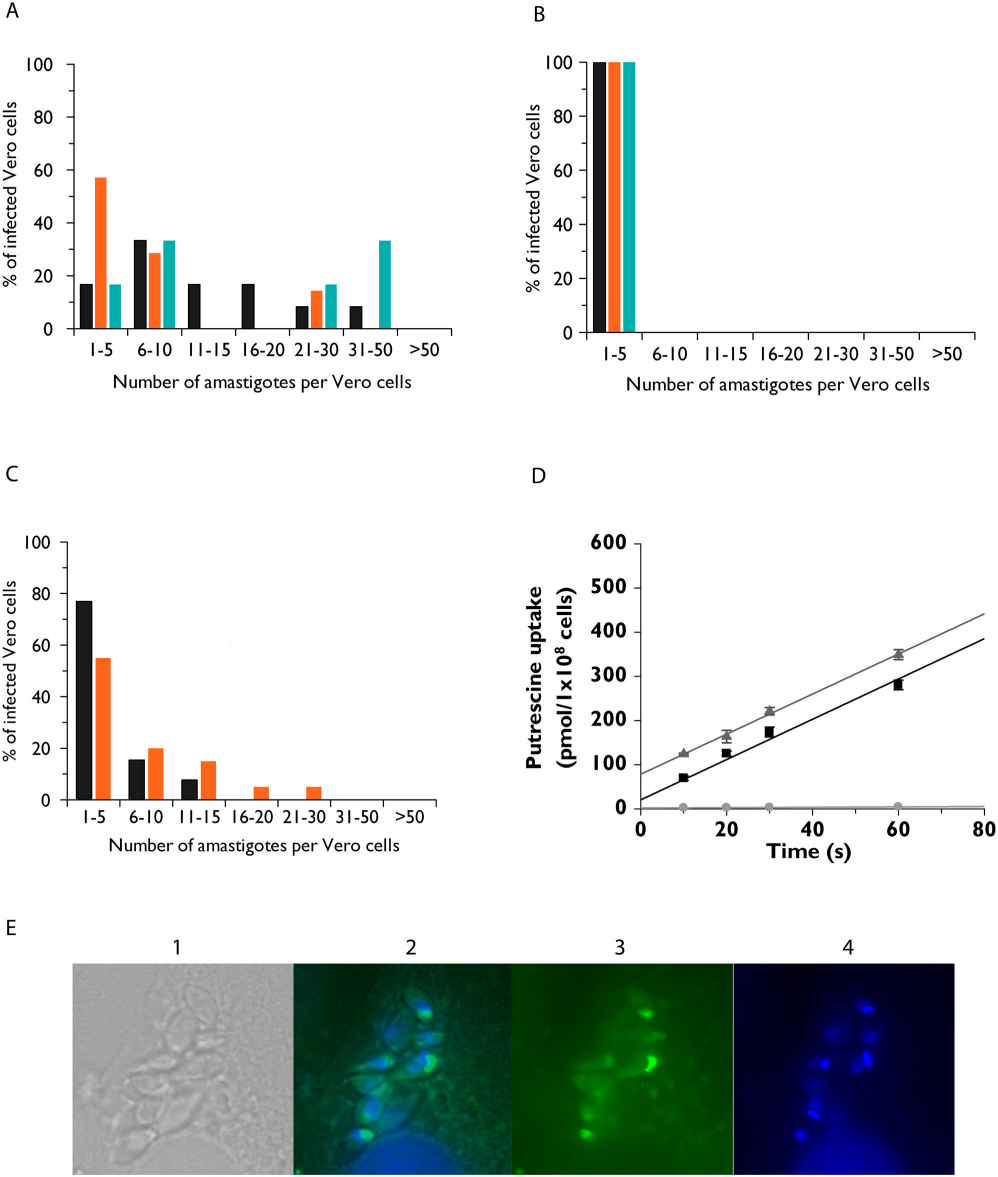
Parasite loads in mammalian cells infected with either wild type, Δ*tcpot1*, or Δ*tcpot1*[p*TcPOT1*] “add-back” parasites. Vero cells were infected with (A) wild type, (B) Δ*tcpot1*, or (C) Δ*tcpot1*[p*TcPOT1*] metacyclic trypomastigotes at a ratio of five parasites per cell and DAPI-stained amastigotes enumerated as indicated in the Experimental procedures section. Each color represents data from three independent experiments for the wild type and Δ*tcpot1* cell lines and is indicated as n1 (black), n2 (orange), or n3 (blue), and data from two independent experiments for the “add-back.” Within each experiment amastigotes enumeration was performed in duplicate. (D) Demonstration that putrescine transport capability has been conferred by ectopic expression of *TcPOT1* in the Δ*tcpot1*[p*TcPOT1*] “add-back” line. Putrescine transport was measured as described for Fig 2 in wild type (■), Δ*tcpot1* (grey dot), and Δ*tcpot1*[p*TcPOT1*::*GFP*] (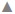) cells, and the data presented are those of three replicates. Direct fluorescence microscopy (x63 magnification) (E) displays expression of *TcPOT1*.*1*::*GFP* in Vero cells infected with *T*. *cruzi* amastigotes. Shown are a phase contrast image (1), a merge of GFP and DAPI images (2), GFP fluorescence (3), and DAPI-stained parasites (4).

To assess whether impaired diamine transport impacted the capacity of *T*. *cruzi* to infect and replicate in mammalian cells, the abilities of wild type and Δ*tcpot1* trypomastigotes to infect Vero cells were compared (Fig 6A–6C). There was no meaningful disparity between the percentages of infected Vero cells exposed to either the wild type or null mutant line, attesting that the loss of the TcPOT1 transporter did not lead to a loss in infectivity of the Δ*tcpot1* cell line (Fig 6). However, there was a significant difference observed in the ability of the Δ*tcpot1* cell line to multiply within Vero cells: while the number of wild type parasites observed per infected Vero cells ranged from 1 to 50 parasites, all Vero cells infected with Δ*tcpot1* parasites contained only 1 to 5 amastigotes (Fig 6A and 6B). A complemented cell line, Δ*tcpot1*[p*TcPOT1*.*1*::*GFP*], was also evaluated for its ability to infect Vero cells. The Δ*tcpot1*[p*TcPOT1*.*1*] cells displayed an intermediate infectivity phenotype with parasite loads that were in-between those obtained for Vero cells infected with wild type or Δ*tcpot1* trypomastigotes (Fig 6C). High affinity putrescine transport capability was restored in the Δ*tcpot1*[p*TcPOT1*.*1*::*GFP*] cells (Fig 6D), and direct fluorescence established *in vivo* expression of the ectopic *TcPOT1* gene (Fig 6E).

Although the *Δtcpot1* lesion had little impact on the growth rate of *T*. *cruzi* epimastigotes, the ability of the intracellular amastigote to proliferate within Vero cells was markedly compromised while the ability to infect Vero cells was not (Fig 6). Thus, the infectious, replicative form of *T*. *cruzi* exhibits a greater requirement for TcPOT1 function than the extracellular, insect vector form of the parasite. This stage-specific divergence on the impact of TcPOT1 on proliferation could have several possible explanations. First, it is possible that the low-affinity, high capacity putrescine uptake system observed in epimastigotes or spermidine and spermine transport is non-operative or dysfunctional in the amastigote. Second, the intracellular environment in which amastigotes reside may be relatively deficient in putrescine content. Although estimates of polyamine levels in mammalian cells and tissues are generally in the millimolar range [46, 47], a large percentage of cellular polyamines is not free in the cytoplasm but, rather, bound to macromolecules such as ATP, phospholipids, and nucleic acids [8, 48]. Moreover, in most mammalian cells, spermidine concentrations are generally an order of magnitude higher than those for putrescine [49–51], and diamine pools may simply be too low to enable proliferation of Δ*tcpot1* amastigotes. Finally, *T*. *cruzi* amastigotes may have a different or greater polyamine requirement than other life cycle stages, and this nutritional necessity cannot be met in the absence of a high affinity putrescine transport system. Consistent with this inference is the observation that spermidine and spermine pools are 12- and 3-fold higher in amastigotes of *T*. *cruzi* than epimastigotes [52].

## Conclusion

The finding that the genetic disruption of the high-affinity diamine transporter, *TcPOT1*, can impact *T*. *cruzi* infectivity sheds light on the delicate balance between polyamine supply and demand that must be sustained for parasite growth within the host milieu. These results also suggest *TcPOT1* could be therapeutically exploitable and bolster the legitimacy of the entire polyamine pathway as a prospective antitrypanosomal drug target for treating *T*. *cruzi* infections. Whether the abridged *de novo* polyamine biosynthetic pathway in which putrescine is converted to spermidine by SPDSYN is also critical for replication awaits genetic analysis using tools similar to those described in this investigation.

## Supporting Information

S1 Fig Gene targeting construct schema based on the Multisite Gateway^®^ Vectors strategy. (DOCX)

S1 Table Sequences of oligonucleotides used to generate the *TcPOT1*.*1* and *TcPOT1*.*2* gene targeting constructs based on the Multisite Gateway^®^ Vectors strategy. (DOCX)
