# Supplementary figures and images for: The Trypanosoma cruzi Diamine Transporter Is Essential for Robust Infection of Mammalian Cells
Source: PLoS One. 2016 Apr 6;11(4):e0152715. doi: 10.1371/journal.pone.0152715 (PMC4822861; doi:10.1371/journal.pone.0152715)

**S1 Fig.**

**
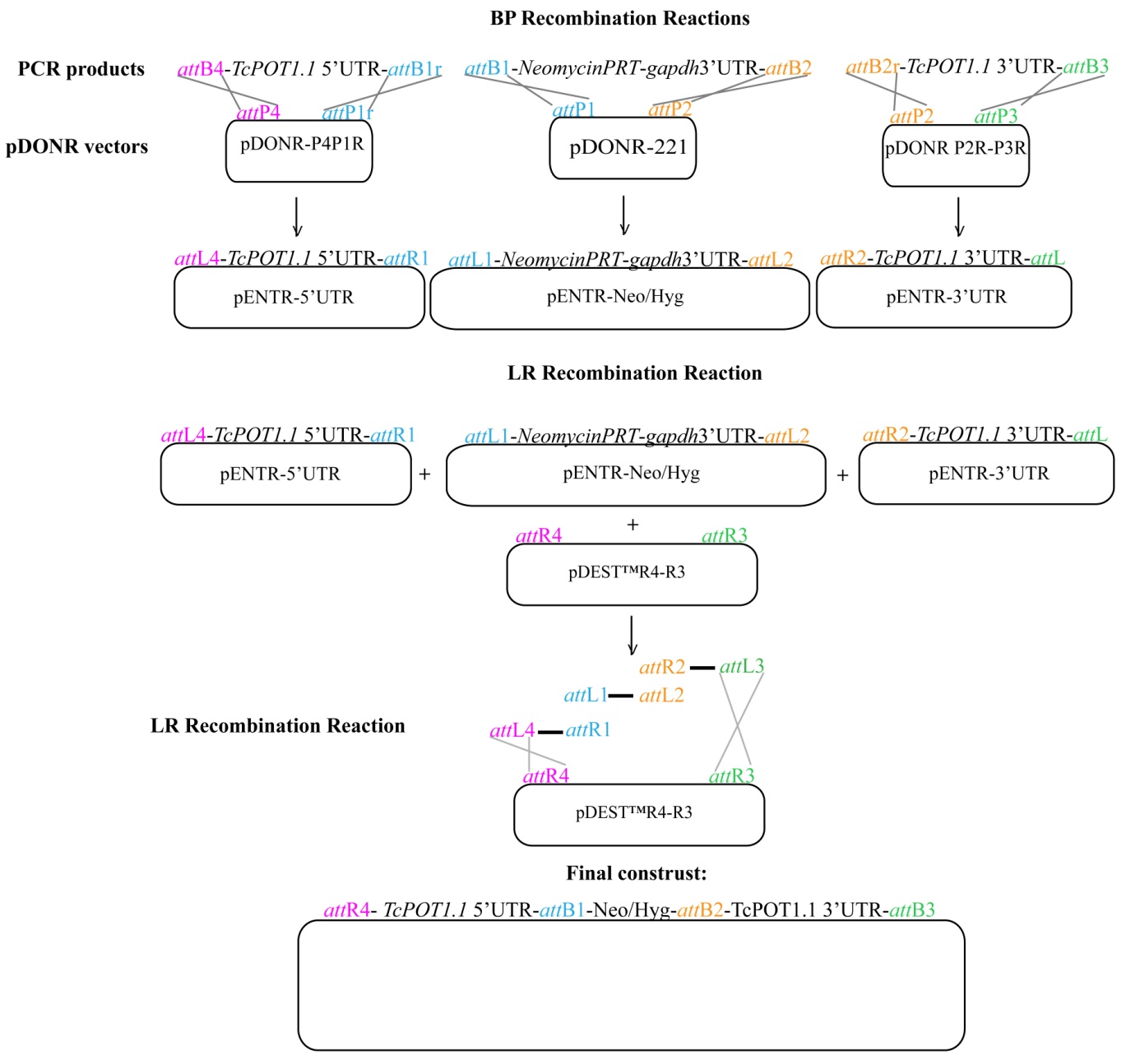
**

Supplement: S1 Fig — (DOCX) [file pone.0152715.s001.docx]
