# Supplementary material for: The Trypanosoma cruzi Diamine Transporter Is Essential for Robust Infection of Mammalian Cells
Source: PLoS One. 2016 Apr 6;11(4):e0152715. doi: 10.1371/journal.pone.0152715 (PMC4822861; doi:10.1371/journal.pone.0152715)
Supplement: S1 Table — (DOCX) [file pone.0152715.s002.docx]

**S1 Table**. Sequences of oligonucleotides used to generate the *TcPOT1.1* and *TcPOT1.2* gene targeting constructs based on the Multisite Gateway^®^ Vectors strategy.

**Primer Name Primer sequence**

*att*B1_Hyg_f GGGGACAAGTTTGTACAAAAAAGCAGGCTATGAAAAAGCCTGAACTCAC

*att*B2_Hyg_r GGGGACCACTTTGTACAAGAAAGCTGGGTCAAATTCTGTTCAATGTAAT

*att*B1_Neo_f GGGGACAAGTTTGTACAAAAAAGCAGGCTATGGGATCGGCCATTGAACA

*att*B2_Neo_r GGGGACCACTTTGTACAAGAAAGCTGGGTCACACGGCTAGCATACTCTA

attB4_5’UTR_TcPOT1.1_f ggggacaactttgtatagaaaagttggcggtcgatgtgaaaaagtac

attB1_5’UTR_TcPOT1.1_r ggggactgcttttttgtacaaacttgaacaacttcccttcctgtcacc

attB2_3’UTR_TcPOT1.1_f ggggacagctttcttgtacaaagtggaacgccggcacctcctgcac

attB3_3’UTR_TcPOT1.1_r ggggacaactttgtataataaagttgcacacacggacagtttacatgc

attB4_5’UTR_TcPOT1.2_f ggggacaactttgtatagaaaagttggtggtcgatgtgaaaaattac

attB1_5’UTR_TcPOT1.2_r ggggactgcttttttgtacaaacttgaacaccttcccttcctgtcaac

attB2_3’UTR_TcPOT1.2_f ggggacagctttcttgtacaaagtggacatcggcggatgaggggtg

attB3_3’UTR_TcPOT1.2_r ggggacaactttgtataataaagttggatttctccctgtgagtagccACG
